# Connected Component Analysis of Dynamical Perturbation Contact Networks

**DOI:** 10.1021/acs.jpcb.3c04592

**Published:** 2023-08-29

**Authors:** Aria Gheeraert, Claire Lesieur, Victor S. Batista, Laurent Vuillon, Ivan Rivalta

**Affiliations:** † Laboratoire de Mathématiques (LAMA), Université Savoie Mont Blanc, CNRS, 73376 Le Bourget du Lac, France; ‡ Dipartimento di Chimica Industriale “Toso Montanari”, Alma Mater Studiorum, Università di Bologna, Viale del Risorgimento 4, 40136 Bologna, Italy; § Univ. Lyon, CNRS, INSA Lyon, Université Claude Bernard Lyon 1, Ecole Centrale de Lyon, Ampère UMR5005, Villeurbanne 69622, France; ∥ Institut Rhônalpin des Systèmes Complexes, IXXI-ENS-Lyon, Lyon 69007, France; ⊥ Department of Chemistry, Yale University, New Haven, Connecticut 06520, United States; # ENS de Lyon, CNRS, Laboratoire de Chimie UMR 5182, 69364 Lyon, France

## Abstract

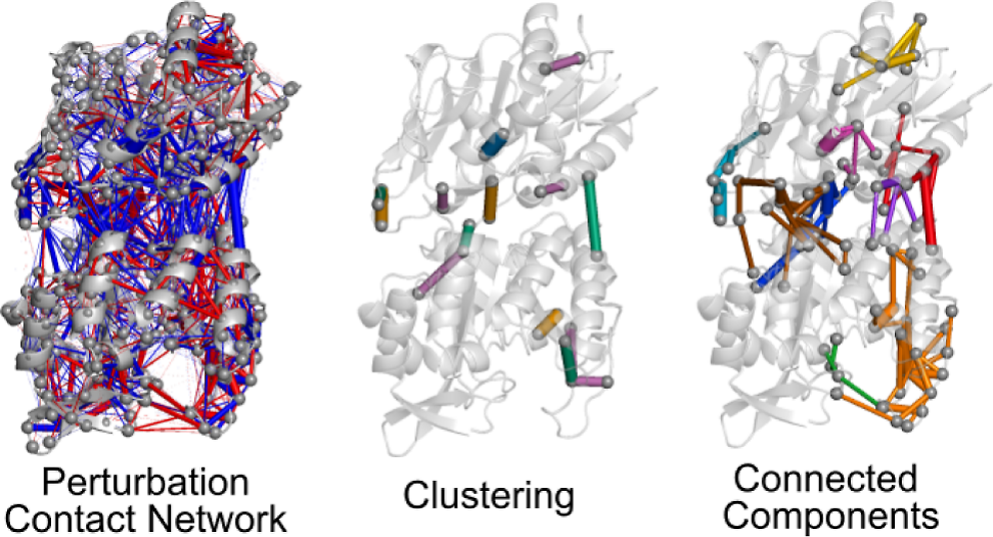

Describing protein dynamical networks through amino acid contacts is a powerful way to analyze complex biomolecular systems. However, due to the size of the systems, identifying the relevant features of protein-weighted graphs can be a difficult task. To address this issue, we present the connected component analysis (CCA) approach that allows for fast, robust, and unbiased analysis of dynamical perturbation contact networks (DPCNs). We first illustrate the CCA method as applied to a prototypical allosteric enzyme, the imidazoleglycerol phosphate synthase (IGPS) enzyme from *Thermotoga maritima* bacteria. This approach was shown to outperform the clustering methods applied to DPCNs, which could not capture the propagation of the allosteric signal within the protein graph. On the other hand, CCA reduced the DPCN size, providing connected components that nicely describe the allosteric propagation of the signal from the effector to the active sites of the protein. By applying the CCA to the IGPS enzyme in different conditions, i.e., at high temperature and from another organism (yeast IGPS), and to a different enzyme, i.e., a protein kinase, we demonstrated how CCA of DPCNs is an effective and transferable tool that facilitates the analysis of protein-weighted networks.

## Introduction

Recent advances in computational technologies have enabled us to perform classical molecular dynamics (MD) simulations on systems of considerably large size and timescale.^1−5^ With such long simulations producing large data sets, understanding the dynamics of the system can be difficult. In recent years, dynamical network analysis has become an increasingly popular and effective computational tool for understanding a variety of biological processes.^6−13^ This approach has been used to examine the arrangement of atoms in proteins^14^ and to study signaling pathways in allosteric systems.^6,8,13,15,16^ A commonly used network approach is the analysis of atomic contacts between amino acid residues in crystal structures of multiple protein types/families.^17−22^ Another approach involves monitoring the contact perturbations induced by mutations,^23,24^ namely, perturbation contact networks. Dynamical networks can be constructed from static networks by running MD simulations and monitoring the frequency of a contact,^25,26^ or the average number of interatomic contacts.^27^ This method has been found to be particularly useful in analyzing the effect of perturbations due to effector binding and thus allosteric signaling mechanisms.^26,27^

The use of dynamical perturbation contact networks (DPCNs) to analyze allosteric signals has enabled a deeper understanding of the local conformation changes that occur during the allosteric regulation of prototypical allosteric proteins, such as the imidazoleglycerol phosphate synthase (IGPS) enzyme from *Thermotoga maritima*.^27^ Compared to more commonly used weighted protein networks, such as those involving weights based on correlated motions obtained from MD simulations,^8,10,28−30^ using dynamical contact networks provides complementary information to acquire a deep knowledge of allosteric signal propagation. This is particularly valid when the allosteric pathways involve the propagation of local contact perturbations from the effector to the active site, as has been demonstrated in IGPS.^8,27,31−33^ In fact, using correlated motions features the limitation of compressing the atomistic details of the signal propagation in a single numerical coefficient for each interacting amino acid pair. Thus, recovering the atomistic information from a protein graph based on correlation coefficients requires the re-analysis of the MD simulations, while the DPCN graph can be easily decomposed in subgraphs for specific types of interactions (e.g., considering only hydrophobic residues or ionic ones, or just backbone atoms to focus on partial folding/unfolding events, etc.), without re-analyzing the MD trajectories. In fact, although various computational means can reach a consensus on allosteric signal propagations, they generally tend to be complementary to each other in the specific aspects under investigation.^34^

By constructing a protein graph weighted by differences in atomic contacts between two systems, such as the apo- and effector-bound IGPS enzymes,^27^ the DPCN can capture allosteric pathways previously described in the literature.^8,32^ Moreover, the DPCN is a powerful tool not only for studying allostery but, in general, can be potentially used for comparisons of MD simulations. This highly transferable method has been demonstrated to be quite useful for studies of various protein dynamics, including the allostery of bacterial IGPS at high temperatures, yeast IGPS, and adenosine monophosphate-activated protein kinase (AMPK), making it a valuable tool.^33,35−37^ In this work, we investigated the capabilities of the connected component (CC) analysis (CCA) tool^38−41^ in providing a fast, robust, and unbiased analysis of DPCNs. The CCA approach can partition the contact network by grouping connected nodes, similar to what has been successfully done for energy-weighted protein graphs,^42^ with the advantage of providing information on the local propagation of the perturbations. In particular, starting by testing the CCA for the DPCN of bacterial IGPS, since it is a well-studied allosteric system, we evaluated its transferability by applying it to IGPS under other conditions (i.e., at high temperature and from another organism) and to a different protein, i.e., to the AMPK allosteric enzyme.

**Figure 1:**
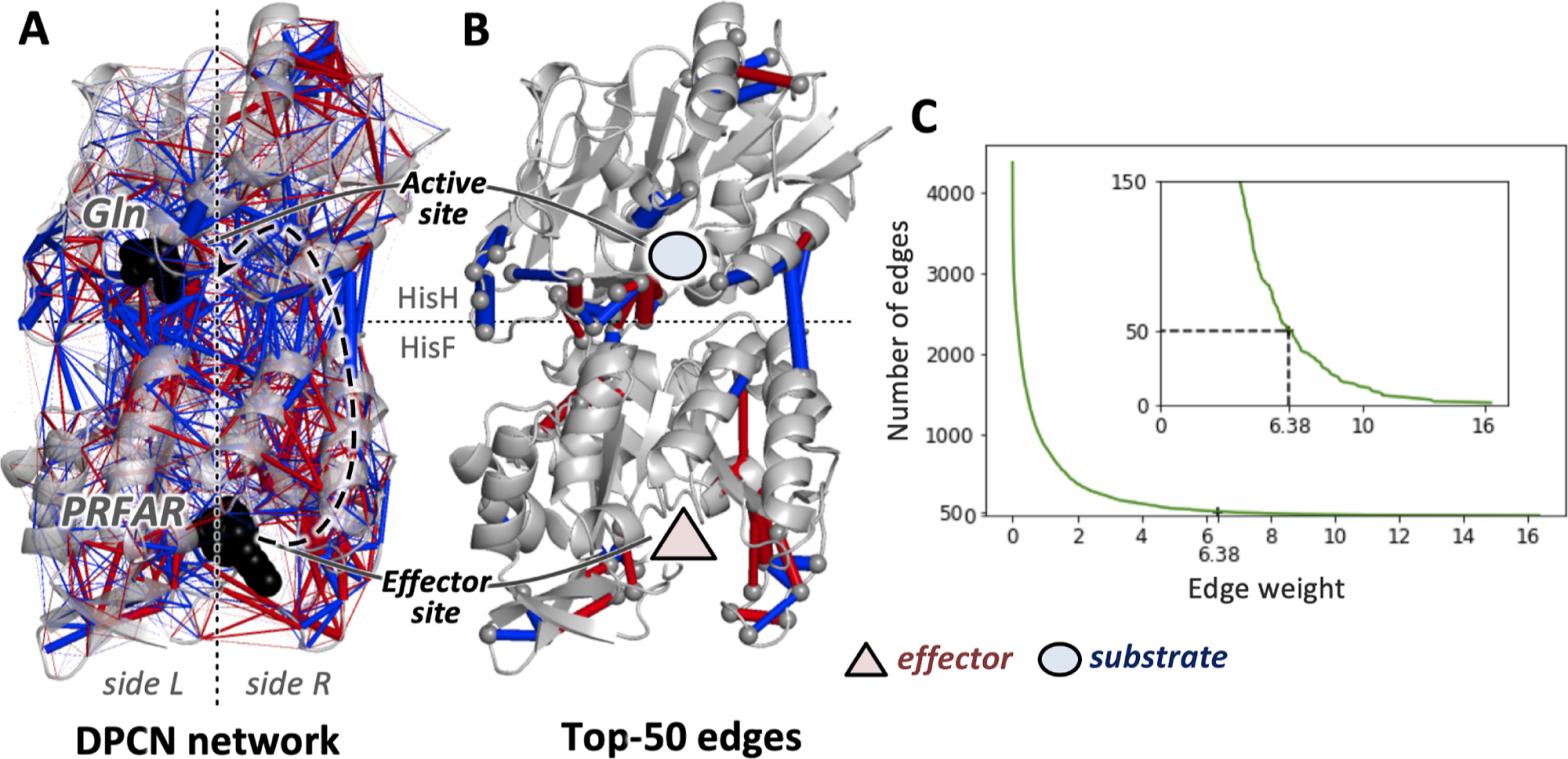
(A) Complete graph associated with the DPCN analysis of PRFAR-bound IGPS and apo-IGPS complexes. Blue edges represent more atomic contacts in apo-IGPS, while red edges represent more contacts in the PRFAR-bound holoenzyme. Binding sites for the effector (PRFAR) and the substrate (Gln) are represented by including the molecular species in black. (B) DPCN graph of IGPS upon removal of edges, leaving only the top 50 edges with the largest weights. Binding sites for the effector and the substrate are represented by a red triangle and blue circle, respectively. (C) Number of edges in the DPCN graph of IGPS decaying as a function of the associated contact weight, with the inset zooming in the region of the DPCN graph shown in panel B.

The IGPS enzyme is composed of two subunits, HisH and HisF. HisH is a glutamine amidotransferase that catalyzes the hydrolysis of glutamine (Gln, the substrate) into glutamate. HisF is a cyclase, where the allosteric effector PRFAR (*N*′-[(5′-phosphoribulosyl)formimino]-5-amino-imidazole-4-carboxamide-ribonucleotide) binds (see Figure 1A). Upon PRFAR binding, the affinity of Gln for HisH increases slightly (fivefold), while the catalytic activity increases by 3 orders of magnitude, making IGPS a V-type allosteric enzyme.^43^ The impressive functionality of IGPS lies in the ability of its active site to detect the allosteric effector over a distance of approximately 25 Å through an allosteric signal that is initiated in the HisF protein and is transferred to the HisH subunit. In a series of computational studies of IGPS allostery,^8,27,31−33^ we have predicted that the allosteric propagation mechanism involves a collection of both short- and long-range displacements, i.e., a set of local (hydrophobic, salt-bridges, and H-bond) interactions that increase the correlations of inter-residue motions on one side of the protein (“side R”, opposite to “side L” indicating right and left sides in the representation depicted in Figure 1A, respectively) and a slow collective motion that alters the HisF/HisH interface, namely, the breathing motion.^8,32^ Our earlier theoretical predictions have been successfully used to design allosteric drugs^31^ and IGPS mutants^44^ that alter the IGPS allosteric pathways, resulting in inactive enzymes. Very recently, both short- and long-range predicted effects have been demonstrated experimentally by X-ray structural characterization of active IGPS ternary complexes^45^ and light-switching activation,^46^ respectively. The secondary structures mainly involved in the IGPS allosteric pathways associated with local contact changes initiated at the effector binding site include elements at the side R of HisF and HisH, which propagate the signal toward the active site, as shown in Figure 1A (see also Figure S1 in the Supporting Information for details of the secondary structure elements involved in these allosteric pathways).

In this context, our DPCN analysis of IGPS allostery has been crucial in discovering the role of specific secondary structure elements and key allosteric contacts. However, this analysis required two arbitrary and biased choices. First, an arbitrary edge weight threshold parameter was chosen (with a brute-force approach) in order to reduce the number of perturbed contacts for an eye-friendly visualization of the perturbed graph. Then, a biased selection of the most relevant perturbations within the reduced network (i.e., the subgraph) was performed based on the previous knowledge of the IGPS allosteric pathways, i.e., focusing on contact perturbations at side R.

Here, we show that by using an unbiased protocol involving a parameter with clear physical meaning, the CCA becomes a powerful tool for the DPCN analysis of MD simulations, showing the successful application to the IGPS allostery under various conditions and transferability to other allosteric proteins.

## Materials and Methods

Aiming to assess the impact of the methodological extension presented here, we started from the DPCN results in ref (27), which introduced DPCN analysis on bacterial IGPS, thus employing previously reported structural models of apo- and PRFAR-bound IGPS complexes from *T. maritima* (described in detail in ref (8)). Accordingly, we used the corresponding MD trajectories that comprise four replica simulations of 100 ns for both the IGPS apoenzyme and the holoenzyme (PRFAR-bound).^8^ Here, a short description (with full details available in ref^8^) of the MD simulations procedure is provided: the AMBER-ff99SB^47^ force field for the IGPS protein and the generalized AMBER force field^48^ for the PRFAR ligand were employed; following a pre-equilibration procedure, production runs were simulated in the NPT ensemble at 303 K and 1 atm, using the Langevin piston, periodic boundary conditions, and particle mesh Ewald method,^49^ as implemented in the NAMD2 software package,^50^ with van der Waals interactions calculated using a switching distance of 10 Å and a cutoff of 12 Å. A multiple time-stepping algorithm^51,52^ was adopted, with bonded, short-range nonbonded, and long-range electrostatic interactions evaluated respectively at every one, two, and four time steps, using a time step of integration of 1 fs.

MD simulations of the yeast IGPS, from *Saccharomyces cerevisiae*, were performed under similar conditions, notably using the same force field. In this system, 12 independent MD simulations of 100 ns (6 for apo and 6 for the PRFAR-bound enzyme) were performed, totaling up to 1.2 μs simulations, with more technical details described in ref (36). During the study of temperature activation of the bacterial IGPS, new simulations were performed using AMBER GPU^53,54^ and the CHARMM36m force field.^55,56^ We generated three replicas for each system, including apo at two different temperatures (30 and 50 °C) and holo at one temperature (30 °C). Each replica was simulated for a duration of 1.5 μs. For the DPCN analysis, we focused on the data from the final microsecond of each simulation. Additional technical details can be found in ref (33). Finally, for the AMPK allosteric system, three replica simulations of 1 μs were performed for each system (apo- and holo-enzymes), using the AMBER ff99SBILDN force field,^57^ with more technical details described in ref (35).

Here, post-processing analyses were performed using the NumPy package,^58^ handling MD trajectories and topologies with MDTraj^59^ and network theory tools of NetworkX.^60^

### Dynamical Perturbation Contact Network

The full procedure to compute the DPCN graph is described in ref (27) and reported here briefly. At each frame of MD simulations, the Cython^61^ implementation of the KD-tree algorithm^62−64^ found in SciPy^65^ is used to get the list of all atomic pairs in contact. From this list, the atomic contact matrix *A*_*ij*_, with elements *a*_*ij*_ = 1 if atoms *i* and *j* are in contact or *a*_*ij*_ = 0 in the opposite case, is constructed. Here, the distance cutoff value of 5 Å was used to define when two atoms are set to be in contact, consistently with the previous analyses.^8,27^ In fact, the 5 Å cutoff value is considered to be a robust choice for protein structure networks, as documented in the literature.^66,67^

The average atomic contact matrix of a set of simulations is defined by averaging each element on all the individual matrices, i.e., . In contrast with atomic contact matrices of each frame, which are binary, the average matrix will generally involve decimal numbers, thus requiring floating-point arithmetic. Finally, this matrix can be converted to the residue contact matrix using transformation matrices *T* such that *t*_*ij*_ = 1 if atom *i* is in residue *j*, or 0 elsewhere. The average residual contact matrix *R* can be expressed as *R* = *T*^*t*^*AT*. This definition allows using different transformation matrices to also describe asymmetric contacts (i.e., contacts between different atomic selections between residues). However, here, the default selection with the protein stripped from hydrogen atoms is used. Looking at intra-residual contacts is beyond the scope of this study; thus, we set all the diagonal elements of the average residue contact matrix to be equal to 0. The average residual contact matrix is then the adjacency matrix of the contact network. The average perturbation contact matrix between an *initial* set of simulations and a *perturbed* one is here defined as the subtraction of their two average residual contact matrices. The DPCN is the network created from the latter adjacency matrix. For visualization purposes, we add a coloring scheme to the edges: blue if the weight is bigger in the *initial* state and red if the weight is bigger in the *final* state.

### Connected Component Analysis

**Figure 2:**
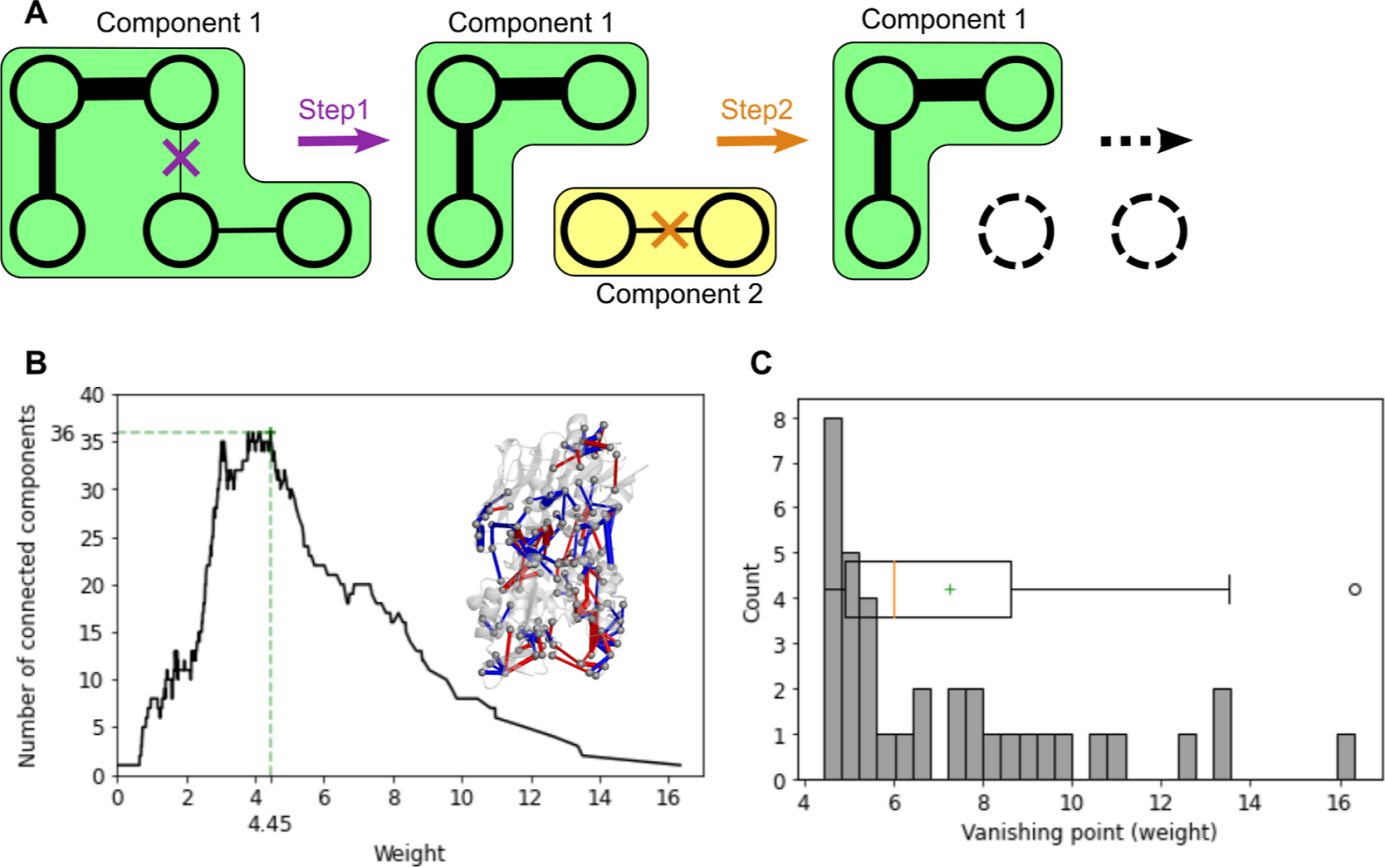
(A) Schematic representation of the changes in the number of components upon sequential removal of edges with the smallest weights. (B) Number of CCs as a function of the contact weight, i.e., the smallest weight left in the graph upon sequential removal of edges. The latest occurrence of the highest number of components, i.e., 36, is highlighted as it is associated with the final CC structure, featuring a minimum edge weight of 4.45. The inset shows such a CC structure in the 3D representation of the IGPS complex. (C) Box plot distribution of the vanishing points of each component in the final CC structure.

Two nodes *u* and *v* in a graph *G* are *connected* if there exists a path in *G* that connects them. The graph *G* is connected if all its nodes are connected, so isolated nodes are not present. The CCs of a graph are the connected subgraphs (so in these subgraphs, there are no isolated nodes) that are not contained in a larger subgraph. In other words, the CCs are the largest subgraphs that are not connected to each other. Thus, a connected graph contains only one CC, which is the whole graph itself, while a unique set of (several) CCs can be defined for disconnected graphs (see Figure 2A). CCs were found, here, using the Breadth-First search algorithm.^68−70^ An amino acid graph representing a protein is generally connected, thus featuring a single CC. However, clearing protein networks of their faintest edges, for instance by using a threshold value to highlight the most relevant connections, is a common practice, making such protein graphs generally disconnected. This is also the case for DPCN graphs,^27,35,37^ for which then the CCs can be generated in order to simplify their analysis and visualization.

## Results and Discussion

The whole graph resulting from a DPCN analysis is quite congested and extremely difficult to be inspected by the human eye. For instance, the DPCN graph obtained by comparison of PRFAR-bound and apo-IGPS MD simulations contains more than 4000 edges, as shown in Figure 1A. From this quite intricate picture, one can still extract some clues about the overall contact changes in the IGPS complex due to effector binding. In fact, a significant amount of contact changes, represented by (colored) edges is clearly visible at the HisF/HisH interface, as a consequence of changes in the relative motion between the two subunits (namely, the breathing motion), which has been shown to be an allosteric effect associated with the effector binding.^8,31−33,44,71^ However, the pattern associated with the most important contribution to the allosteric pathways, which is running along one side of IGPS (namely, side R), is difficult to detect in the complete DPCN graph since the visual inspection of a large number of edges represents a quite tedious work.

In order to obtain DPCN subgraphs whose graphical representations are both easy to visualize and featuring patterns associated with the allosteric pathways, a reliable way to remove/select edges from the complete DPCN graphs must be determined. In previous studies,^27,35,37^ we decided to remove edges below a certain threshold weight from the complete DPCN graph or from some of its subgraphs, such as those resulting from specific contacts among backbone atoms, or heavy atoms (removing hydrogens), etc. Notably, for each type of graph, a certain threshold weight was chosen, based on various attempts of graph visualization. Such arbitrary choices call for more reliable ways of edge selection in DPCN analysis. Considering what a human eye can feasibly analyze from a representation of a DPCN graph, one could first consider a *brute-force* approach in which just the 50 edges with the largest weights, i.e., representing the largest changes in number of contacts, are left in the subgraph. For the DPCN of IGPS, this will correspond to the choice of a threshold weight equal to 6.38, resulting in the graph depicted in Figure 1B. With such crude selection criterion, one can visualize the HisF perturbations near the effector site at side R; partial propagation toward the active site, and also several other displacements (at sideL, at the HisF/HisH interface, and at the top of HisH) can be detected. This *brute-force* approach, thus, does not really fit the wish to selectively recognize the allosteric pathways at side R and, anyway, it involves an arbitrary selection of the number of edges (i.e., 50 in this example).

Therefore, one can attempt to use clustering techniques for the selection of DPCN subgraphs that detect allosteric pathways. In the Supporting Information, we report an extensive analysis of such attempts. In particular, we evaluated the clustering of the edges according to their weights, which are related to the number of contact perturbations, i.e., with edges in the same cluster representing similar perturbations. Then, we ranked these clusters going from largest to smallest weights and selected the most important ones. This approach provided interesting results in terms of recognizing relevant contact perturbations but suffered from two main limitations: i) this analysis does not provide insights on the local propagation of contact perturbations and, thus, straightforward detection of allosteric pathways and ii) the choice of the number of important clusters to take into consideration remains rather arbitrary and potentially biased by previous knowledge of the allosteric signaling mechanism.

### Connected Component Analysis

Given the limitation of the *brute-force* and clustering approaches discussed above, we introduced CCA as a tool that provides a way to cluster edges by spatial proximity, possibly granting information on the local propagation of allosteric signals. As shown in Figure 2A, when sequentially removing edges with the smallest weights, the number of CCs (after pruning isolated nodes) can increase (if this edge is the last one connecting two components, as in Step 1 of Figure 2A), decrease (if the edge is the last member of a component; see the disappearance of component 2 in Step 2 of Figure 2A), or plateau (if the edge is inside a CC with more than one edge). The DPCN graph of IGPS is “connected” in the sense of a topological space, i.e., there is a path from any node to any other node in the graph (see Figure 1A); thus, it contains just one CC. This initial, single CC is conserved until the edges with weights <0.80 are removed, as shown in Figure 2B. Then, the first split of the DPCN graph into two CCs occurs, and the number of CCs steadily increases until around 35 (for weights equal to 3.03). This means that in the range of 0.8–3.03, the action of removing an edge generally creates a new component. Then, moving to the 3.03–4.45 range, the number of CCs oscillates between 30 and 36 components. Here, the components are created and destroyed approximately at the same rate, thus featuring fast and small oscillations for sequential edge cuts. The network with the maximum number of components at the largest weight (i.e., at 4.45) is considered as the graph containing its “final” components, i.e., the final CC structure, since from this point, the number of components is destined to quickly decrease after each edge cut. Indeed, only occasionally, the number of components can slightly increase upon a new edge cut after this point, but with the total number of components always smaller than the maximum (36, found at weight 4.45) (see Figure 2B). From this point, in fact, edge removal will create mostly (pruned) isolated nodes, indicating that a graph structure where the components are interconnected by edges with large weights is reached. This structure resembles a community structure, where the edges with the smallest weights are removed and the corresponding nodes pruned. Indeed, as shown in Figure 2B, there is a fast decrease in the number of CCs from 36 to 20 between weights 4.45 and 6. This corresponds to the removal of the smallest components, usually containing a single edge. At weights >6, the number of components undergoes a much slower decay (even plateauing in some ranges) until zeroing at the final weight of around 16. This behavior is due to the strongest components that disappear slowly. Here, then, the strength of a given component is related to its edge with the largest weight, namely, the “vanishing point” of that component.

Figure 2C shows the distribution of vanishing points for the final CC structure (with 36 components). About 50% of these components have a vanishing point between 4.45 and 6, which belongs to the initial fast decrease in the number of components down to 20. The median value of the whole distribution of vanishing points is 6.01, which, notably, is quite close to the threshold weight corresponding to the top 50 edges (i.e., 6.38). Above 6, the distribution of vanishing points is really spread, with one or (maximum) two components sharing the same vanishing point, in line with the slow disappearance of components following edge removal with weights >6, as discussed above.

The number of edges and nodes, namely, the size and order of the components, respectively, in the final CCs (see Figure S7 in the Supporting Information) shows that the sizes of the 36 CCs are typically quite similar to their orders. This implies that the CCs are not a fully connected subgraph (i.e., not all edges are connected with each other), but they are relatively sparse and spread along the DPCN graph. Another interesting metric for classifying a CC is its diameter (*d*), defined as the largest distance (i.e., the number of edges in the shortest path) between any pair of nodes present in the component.

**Figure 3:**
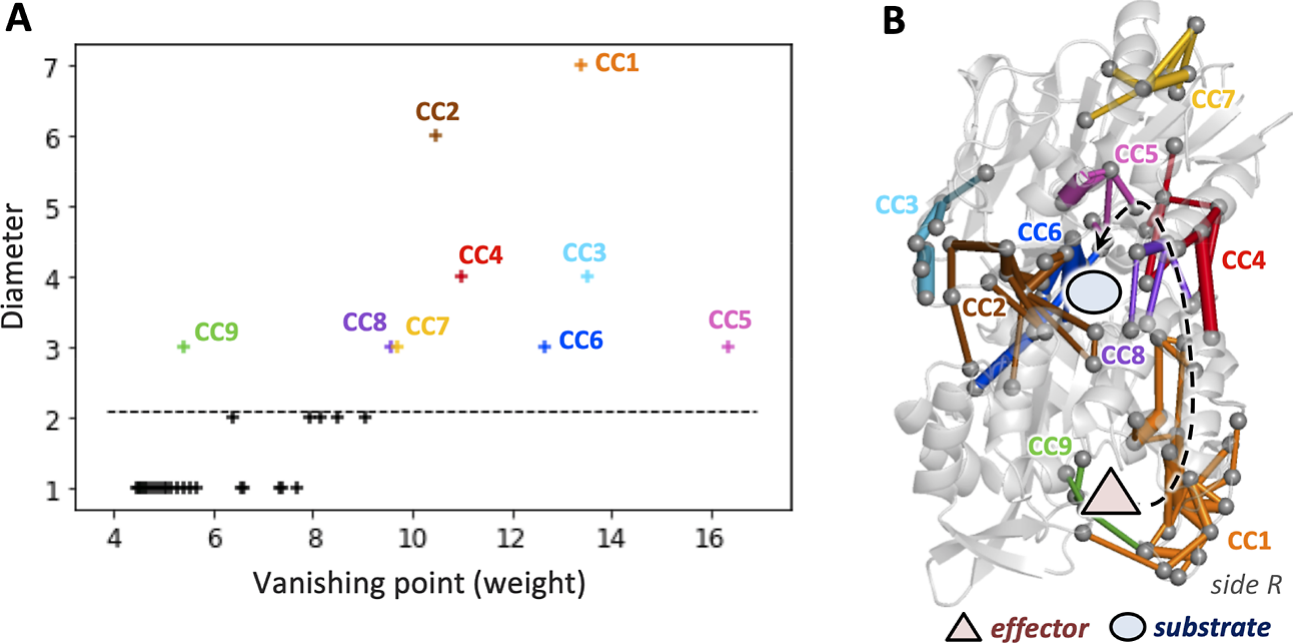
(A) Scatter plot of the relation between the diameter of the CCs in the final CC structure and their vanishing points, with colored points assigned to CCs with *d* > 2. (B) DPCN containing the major CCs (with *d* > 2) plotted in the 3D structure of IGPS, with a color scheme following the scattered plot in (A). Binding sites for the effector and the substrate are represented by a red triangle and blue circle, respectively. The black arrow indicates the allosteric pathways associated with the local propagation of contact perturbations from the effector to the active site.

For the DPCN graph analyzed here, the sizes of the final components indicate how much influence a local perturbation has on other amino acids, or, better, how many amino acids are impacted by the perturbation spreading. On the other hand, the diameter evaluates how far the perturbation is spread through the protein matrix. Notably, we observed an interesting trend of the diameters of the final components as a function of their vanishing points, which is depicted in Figure 3A. Most of the 36 final components are composed of two nodes and one edge, so they have a diameter of 1, but also a good portion of them have a diameter of 2 and represent the case of the first (minimal) propagation of the contact perturbations. The overall distribution of the diameters of the final components is reported in Figure S8 in the Supporting Information. Notably, as reported in Figure 3A, the CCs with the highest vanishing points also feature the highest diameters. As we are interested in exploiting the CCA to characterize the local propagation of allosteric signals, it is remarkable to observe that the components with the largest vanishing points, i.e., containing as a maximum edge weight a large value (associated with many atomic contacts perturbed), likely feature large diameters. This implies that the pair contact perturbations are not just many within these CCs, but they also spread significantly across the protein graph.

Thus, in order to analyze the allosteric signal propagation detectable by the DPCN analysis of IGPS, we focused on the most relevant CCs of the final CC structure, i.e., the CCs featuring a diameter associated with a sizable spreading of the contact perturbations (*d* > 2). This choice is, in fact, not really arbitrary as it has a clear physical meaning, implying that one considers just the cases that are beyond the minimal (*d* = 2) propagation of the contact perturbations. As shown in Figure 3B, this choice selects 9 CCs, spreading across the entire IGPS protein complex but, notably, now recovering the allosteric pathways of IGPS expected to run along the side R. These CCs involve specific secondary structures, which are listed in Table S1 in the Supporting Information. The main advantage of the CCA proposed here is the rational partitioning and automatic drawing of a DPCN subgraph, which allows for an easy understanding of the local contact perturbations associated with allostery. In fact, in reference,^27^ we used a brute-force approach to analyze the DPCN graph, employing a set of different weight thresholds and various selections of atoms that define the contact subgraphs. Such tedious work can be avoided if the proposed CCA is adopted, facilitating the application of the DPCN method to much larger and more complex systems compared to IGPS.

### Applications of CCA to Other Proteins and Conditions

**Figure 4:**
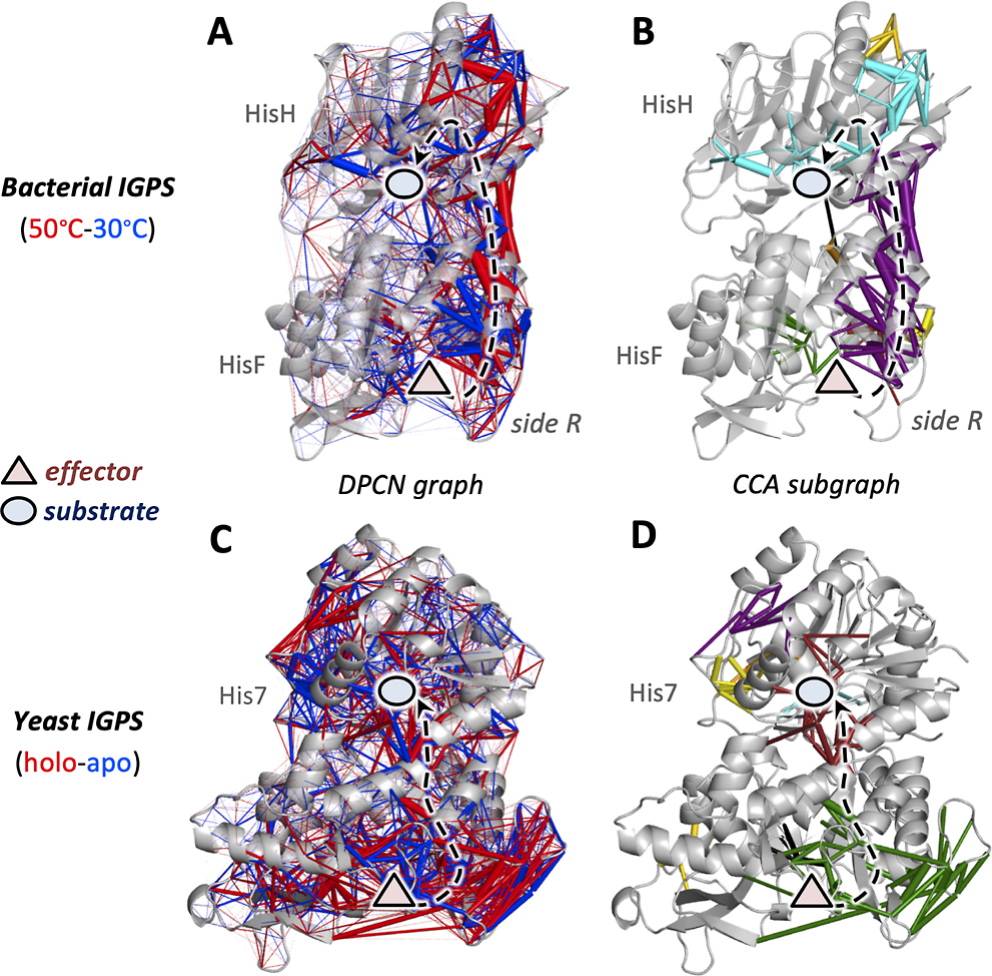
Complete DPCN graphs and the corresponding CCA subgraphs for the allosteric signal induced by temperature increase in bacterial IGPS (A,B) and effector binding in yeast IGPS (C,D). Binding sites for the effector and the substrate are represented by a red triangle and a blue circle, respectively. The black arrow indicates the allosteric pathways associated with the local propagation of contact perturbations from the effector to the active site.

After showing that the CCA approach can effectively detect the propagation of the atomic contact perturbations associated with the allosteric pathways of bacterial IGPS, in the following, we discuss the application of this approach to other proteins and conditions. In particular, in our previous investigations, we demonstrated the peculiar effects that temperature has on the allosteric pathways of bacterial IGPS.^33^ The temperature increase significantly alters the collective motions associated with the IGPS allostery but, unexpectedly, it also triggers a cascade of local contact perturbations (probed with our DPCN approach) that remarkably resembles the allosteric activation induced by the effector binding, i.e., the allosteric pathways along side R of the IGPS complex. Since it was experimentally proved that the temperature increase mimics the allosteric activation of IGPS by the effector binding,^43^ this further demonstrates the role of local contact perturbations in the IGPS allostery. Therefore, we used the CCA protocol proposed here to verify if this approach can also provide a fast and robust tool for the detection of temperature-dependent allosteric pathways. As shown in Figure 4A,B, the CCA partitioning of the complete DPCN graph, computed by subtracting the protein contacts network at 50 °C from that at 30 °C, indeed provides clear evidence of the side R allosteric propagation of local contact perturbations induced by the temperature increase.

On the other hand, one might wonder if such perturbations of local contacts among amino acid residues, equally induced by effector binding and temperature increase, are “conserved” in the protein family of the IGPS enzymes. To address this question, we previously investigated the allosteric pathways in the IGPS enzyme from another organism, in particular from the *S. cerevisiae* yeast.^36^ We found rather distinct allosteric pathways between the IGPS from the yeast with respect to those of the bacteria, in terms of both collective motions and local contact perturbations. The latter, in particular, were determined by our DPCN method and indicated that by partially changing the primary sequence of the IGPS enzyme, going from bacteria to yeast, the allosteric signal propagation (induced by effector binding) shifts from the surface residues on side R to pathways located more internally in the protein matrix, respectively. As shown in Figure 4C,D, in fact, the yeast IGPS features a single protein chain, namely, His7, in contrast with the HisF/HisH heterodimeric structure in the bacteria, and it has a very similar, but not identical, protein scaffold. In the case of yeast IGPS, the DPCN complete network, computed by subtracting the protein contact network of the holoenzyme (associated with PRFAR binding as in bacterial IGPS) with that of the apoenzyme, features numerous contact perturbations (see Figure 4C) that are, as expected, very difficult to disentangle by visual inspection. Here, the application of our CCA protocol (see Figure 4D) nicely provides a clear picture of the allosteric pathways that, as mentioned above, are not anymore associated with the surface-exposed residues at side R, as shown in bacterial IGPS for both effector binding (see Figure 2B) and temperature increase (see Figure 4A,B). In fact, the CCs selected in the DPCN of yeast IGPS connect the effector and the active sites via local contact perturbations that lie in the inner part of the IGPS protein (see Figure 4D).

**Figure 5:**
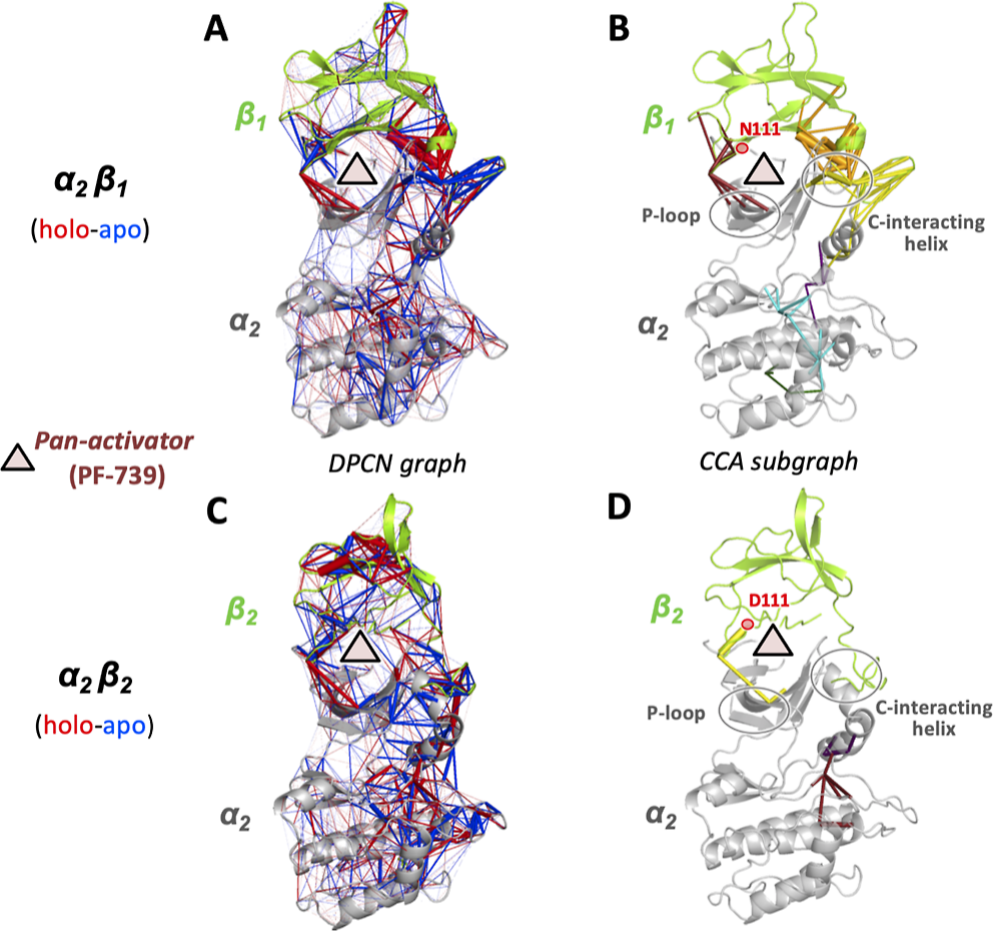
Complete DPCN graphs and the corresponding CCA subgraphs for the allosteric signal induced by binding of the PF-739 pan-activator to the α2β1 (A,B) and α2β2 (C,D) different isoforms of AMPK. Blue edges represent more atomic contacts in apo AMPK, while red edges represent more contacts in the activator-bound holoenzyme. The ADaM binding site for the pan-activator is represented by a red triangle, while gray circles highlight the secondary structure elements of the α2 subunit interacting with the β ones. The N111 and D111 residues differentiating the β1 and β2 subunits are indicated in red.

Finally, in order to further demonstrate the transferability of the CCA approach to other types of allosteric protein, we applied it to our DPCN study of the AMPK enzyme.^35^ AMPK is an energy sensor that has a fundamental role in regulating cell metabolism and thus is often used as a target for metabolic diseases. It is a heterotrimeric complex, consisting of a catalytic α-subunit and two (β and γ) regulatory subunits, which are finely regulated by different mechanisms. In fact, each of these subunits can be found in different isoforms, i.e., α1, α2, β1, β2, γ1, γ2, and γ3, whose selectivity toward specific AMPK activators can be significantly different.^72^ In our recent work,^35^ we were able to characterize key features that mediate the different activation of the α2β1 and α2β2 isoforms induced by the so-called “pan-activators” (in particular, two molecules named with the codes PF-739 and A-769662) that bind in the allosteric drug and metabolite (ADaM) site of AMPK (see Figure 5).

One of the main conclusions of our study was that the subtle difference in the two β isoforms, featuring different residues in position 111, i.e., N111 and D111 in β1 and β2, respectively, can change the networks of atomic interactions in the ADaM site, ultimately providing a better mechanical response of the α2β1 isoform toward the interaction with the pan-activators than that observed in the case of the α2β2 species.^35^Figure 5A,C shows the changes in local contact perturbations due to the binding of the PF-739 pan-activator in the ADaM site, as obtained with our DPCN method. Looking at the complete DPCN graphs, many perturbations are observed across the α2β1 and α2β2 complexes, including those nearby the ADaM site. However, applying the CCA protocol proposed in this work, the significant differences between the isoforms with regard to the propagation of the local contact perturbations appear quite clear. In fact, contrary to what it appears from a first look at the complete DPCN graphs (see Figure 5A,C), the CCA subgraphs shown in Figure 5B,D clearly demonstrate how the subtle difference at the 111 position significantly changes the number of selected CCs, being much larger in α2β1 than in α2β2, featuring 6 and 3 CCs with *d* > 2, respectively. Moreover, considering the secondary structure elements of the α subunit interfacing with the β ones, i.e., the so-called P-loop and C-interacting helix, the CCA results clearly show that the number of local contact perturbations in the selected CCs is also significantly larger in α2β1 than in α2β2. These results are in line with the experimental evidence of better activation response for the α2β1 isoform with respect to the α2β2 one. Moreover, they clearly highlight both the effectiveness and the transferability of the proposed CCA analysis for the study of signal propagations in proteins.

## Conclusions

Protein graphs can be used to provide simple representations of dynamical chemical interactions within complex proteic systems, which are particularly useful for understanding allostery. DPCN graphs, representing connections (i.e., edges) among pairs of amino acid residues weighted by the number of atomic contacts, have been previously proven to be valuable networks for the analysis of signal propagation in prototypical allosteric proteins. The visualization of dense DPCN graphs, however, requires a reliable selection of the protein network for catching the regions that are relevant to the process under study, i.e., the allosteric pathways associated with local contact perturbations. The simple selection of a limited number of perturbed atomic contacts, e.g., 50 edges, represents an arbitrary but still reasonable choice for the primary visual inspection of a DPCN graph. Such selection, indeed, corresponds to an edge weight threshold of about six atomic contacts, which was previously chosen for the DPCN analysis of bacterial IGPS. Still, besides being arbitrary, this *brute-force* solution would not be transferable to other proteic systems since for a given edge threshold, the number of selected perturbed contacts would significantly change with the system size.

On the other hand, clustering methods could be employed to automatically select groups of perturbed edges with similar relevance in the network, i.e., with similar weights. This provides a less arbitrary and more transferable method than brute-force edge selection. However, the clustering of the weighted graph is performed in the edge weight space rather than in the three-dimensional space of the proteic system, hampering the direct detection of the local propagation of perturbations that characterizes the allosteric pathways. Moreover, the clustering approach gives a way to progressively increase the dimension of the selected subgraph by defining the number of clusters to be considered, which still remains somehow arbitrary and system-dependent.

Here, we propose a CCA protocol to analyze weighted DPCN graphs, minimizing the bias and the arbitrary selection of parameters. We used the bacterial IGPS as the reference allosteric system, and we tested the transferability of this tool by analyzing its performance for other proteins and conditions. We showed that CCA is a powerful tool that clusters the DPCN graph, preserving spatial proximity, and provides an automatic selection of the perturbations associated with the allosteric pathways of bacterial IGPS. Here, the only parameter that has to be selected is the component’s diameter *d* that represents just the degree of expansion of the local propagation that one wants to consider and, thus, it is system independent and with a clear physical meaning. Indeed, excluding the trivial cases of single-pair interactions (with *d* = 1) and of minimal propagation (*d* = 2), we observed that the structure of CCs with *d* > 2 for the DPCN of IGPS involves nine components that properly describe the allosteric propagation in terms of atomic contact perturbations. In fact, besides localizing the main effector perturbations, as expected, the CCA is also able to straightforwardly capture the allosteric contacts that previously required fine-tuning of DPCN parameters. Finally, the proposed CCA protocol has been tested for detection of the allosteric pathways in the bacterial IGPS at high temperatures, in the yeast IGPS, and in the activation of AMPK enzymes. These successful tests demonstrated the transferability and the robustness of this approach, which would greatly facilitate the automatic analysis of any perturbation contact networks. Since this tool is easy to implement and applicable to all weighted networks, the proposed method features the potential to become a standard procedure to guide the investigation of different types of protein-weighted networks.

## Supporting Information Available

Detailed allosteric pathways of IGPS, additional results including various edge clustering methods, Birch clustering, different representations of groups of relevance, and distribution of size, order, and diameter of the final components (PDF)
