# Supplementary material for: Connected Component Analysis of Dynamical Perturbation Contact Networks
Source: J Phys Chem B. 2023 Aug 29;127(35):7571–80. doi: 10.1021/acs.jpcb.3c04592 (PMC10493978; doi:10.1021/acs.jpcb.3c04592)
Supplement: Supplementary file 1 — jp3c04592_si_001.pdf [file jp3c04592_si_001.pdf]

Supplementary Information to Connected Component Analysis of Dynamical Perturbation Contact Networks

Aria Gheeraert,[†,‡] Claire Lesieur,[¶,§] Victor S. Batista,[‖] Laurent Vuillon,[*,†,§] and Ivan Rivalta[*,‡,⊥]

†*LAMA, Université Savoie Mont-Blanc, CNRS, Bourget-du-Lac, France*

‡*Dipartimento di Chimica Industriale "Toso Montanari", Università di Bologna, Bologna, Italy*

¶*University of Lyon, CNRS, INSA Lyon, Université Claude Bernard Lyon 1, Ecole Centrale de Lyon, Ampère, UMR5005, 69622 Villeurbanne, France*

§*Institut Rhônalpin des systèmes complexes, IXXI-ENS-Lyon, 69007 Lyon, France*

‖*Department of Chemistry, Yale University, New Haven, Connecticut, 06520, USA*

⊥*ENSL, CNRS, Laboratoire de Chimie UMR 5182, 46 allée d'Italie, 69364 Lyon, France*

E-mail: laurent.vuillon@univ-savoie.fr; i.rivalta@unibo.it

Phone: +33 4 79 75 87 33; +39 051 209 3617

IGPS allosteric pathways

The secondary structures mainly involved in the IGPS allosteric pathways, as shown in Figure S1, initiated at the effector binding site, include elements at the sideR of HisF (*loop1*, $f\alpha1$, $f\alpha2$, $f\alpha3$, $f\beta1$, $f\beta2$, and both $f\alpha2$-$f\beta3$ and $f\beta8$-$f\alpha8$' turns) and HisH ($h\alpha1$ and $h\alpha4$), which propagate the signal through the Ω-loop towards the active site, where the so-called oxyanion strand (i.e. the conserved 49-PGVG sequence) undergoes as significant conformational change assisted by the partial unfolding of the $h\alpha2$ helix.

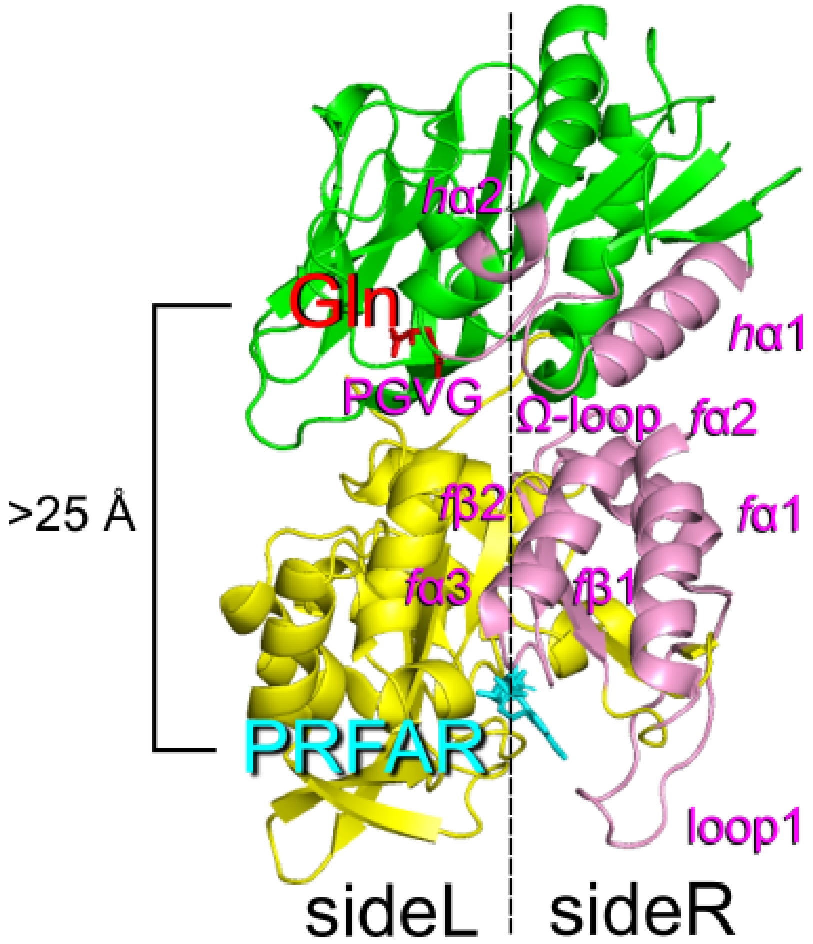

Figure S1: Allosteric mechanism of IGPS from *Thermotoga maritima*. The substrate (glutamine) is positioned in the active site and represented in red. The effector (PRFAR) is positioned in the effector site and represented in cyan. HisF is in yellow and HisH in green. Key secondary structure elements are represented and labeled in pink.

Birch Clustering

To cluster the weighted network, we represented the list of absolute edge weights in the DPCN as a one-dimensional vector. On this vector we performed the scikit-learn[1] implementation of Birch clustering,[2] with a threshold of 0.5 and a branching factor of 50. In order to bypass an arbitrary choice of a number of clusters, we generally did not perform the final clustering step, except when mentioned. For the other clustering methods that have been used for comparison with Birch's one, we employed standard hyperparameters of scikit-learn version 1.1.2.

Clustering and groups of relevance

An analysis of the edge counting decay as a function of contact weight showed in Fig. 1C in the main text reveals that the choice of an arbitrary number of edges (ranked by weight), such as the top-fifty chosen above, is arbitrary and arguable, because it will not necessarily preserve groups of edges that have very similar weights. In other words, the edges ranked right below position 50, e.g. 51-55, might have contact weights very similar to those of the latest edges in the top-fifty list and be relevant spots in the graph. On the other hand, choosing a general threshold weight cutoff is also arbitrary and, however, does not guarantee a human-friendly selection of edges. Indeed, choosing for instance a threshold weight of 5.50 in the DPCN graph of IGPS would leave 75 edges in the network, increasing the number of edges by 50% (with respect to the top-fifty) despite the small variation in weight threshold (with respect to 6.38).

In order to identify groups of edges with similar contact weights, named *groups of relevance*, we employed clustering methods, which provide a set of edges groups that can be ranked by their relevance in the weighted network, i.e. by the amount of contact perturbations that they incorporate. We tested several clustering techniques applied to the DPCN graph of IGPS, as shown in Figure S4. Among them, the Birch clustering resulted to be the most suitable technique for the DPCN network, as explained below.

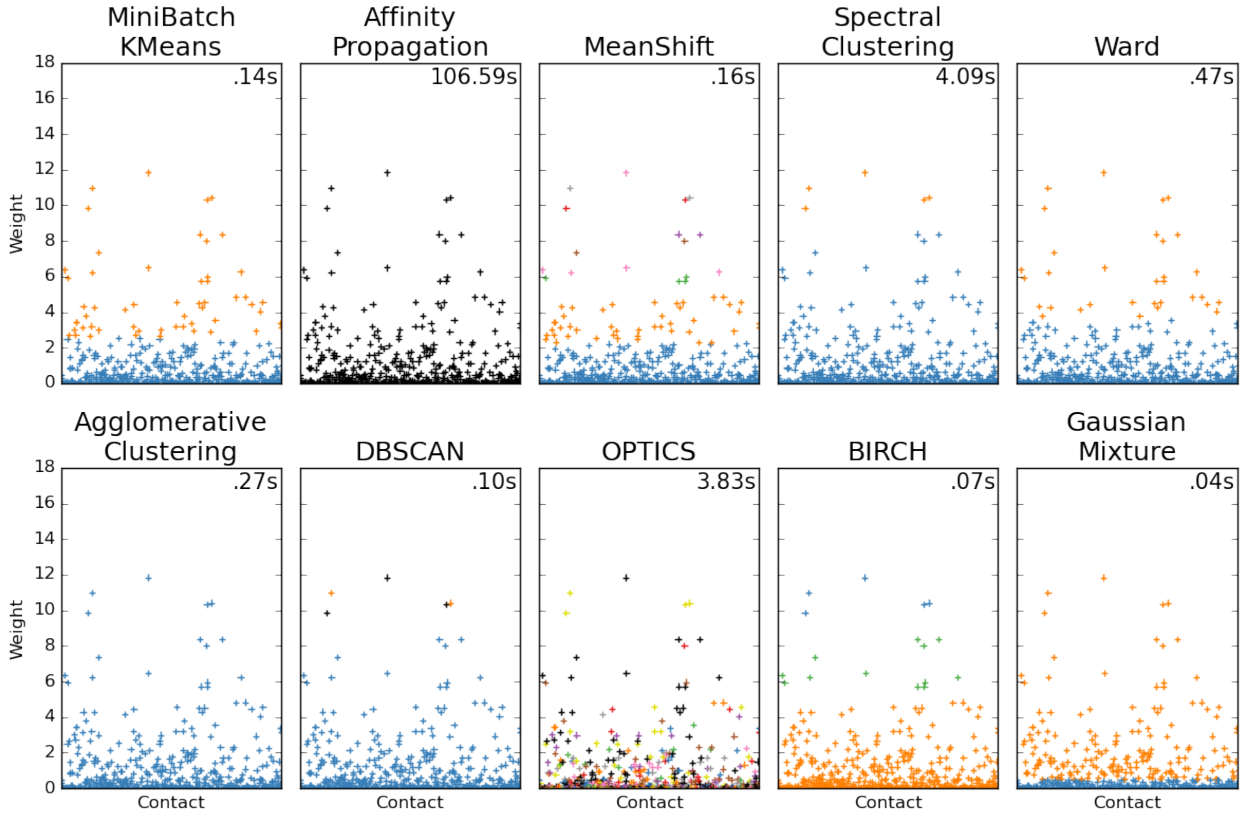

Figure S2: Clustering techniques with different colors indicating the clusters in which each data point belong. The time taken for each clustering is written in each plot. For algorithms asking specifically for a number of clusters, we chose 2 clusters in each case and for algorithms asking for a number of neighbors, we chose 10 neighbors in each case. The running time of each algorithm is indicated in the upper right part of each panel.

Figure S3 shows the portion of DPCN graph associated with edges in the top-four groups of relevance, used as example (with other cases reported in Figure **??**). The group with the highest relevance, i.e. the largest contact perturbation weight, contains a single pair, i.e. the contact hE56-hR59 that is involved in the unfolding of the h2 helix of HisH. The second group of relevance involves three edges: fA224-fF227, hR116-hD159, fR249-hY136. The first pair has been attributed to a displacement of a hydrogen-bonding near the effector binding site,[3] marking the beginning of the allosteric pathways, while the two other interactions are related to the breathing motion in a direct, with fR249 that is part of the so-called molecular hinge of the HisF/HisH interface,[4] or indirect way, with hR116-hD159 being a contact pair in HisH

affected by the interface motion. The third group of relevance contains four edges: fD11-fK19, fE67-hR18, hS115-hD159, fK4-fV248. The first two are recognized key elements of the allosteric mechanism,[4] the third one is directly related to the hR116-hD159 perturbation discussed above, while the latter one involves the connection between the ion gate (i.e. fK4) and the breathing motion (i.e. fV248 is adjacent to fR249 of the molecular hinge). The fourth group contains six edges (including a triad): fK4-fF214, hH120-hH141, fH228-fK19-fR27, hN12-hN15 and hY79-hS197. Among these six edges, the first one is associated to the opening of the ion gate (i.e. fK4) as well as the second one (being adjacent to hM121),[3] while the triad, involving *loop1* (fK19 and fR27) in the effector binding site, and the hN12-hN15 pair have been recognized as part of the allosteric signaling mechanism.[4] The latter perturbation of this group has not been previously highlighted, since it belongs to a cluster of interaction in HisH not directly linked with other relevant perturbations.

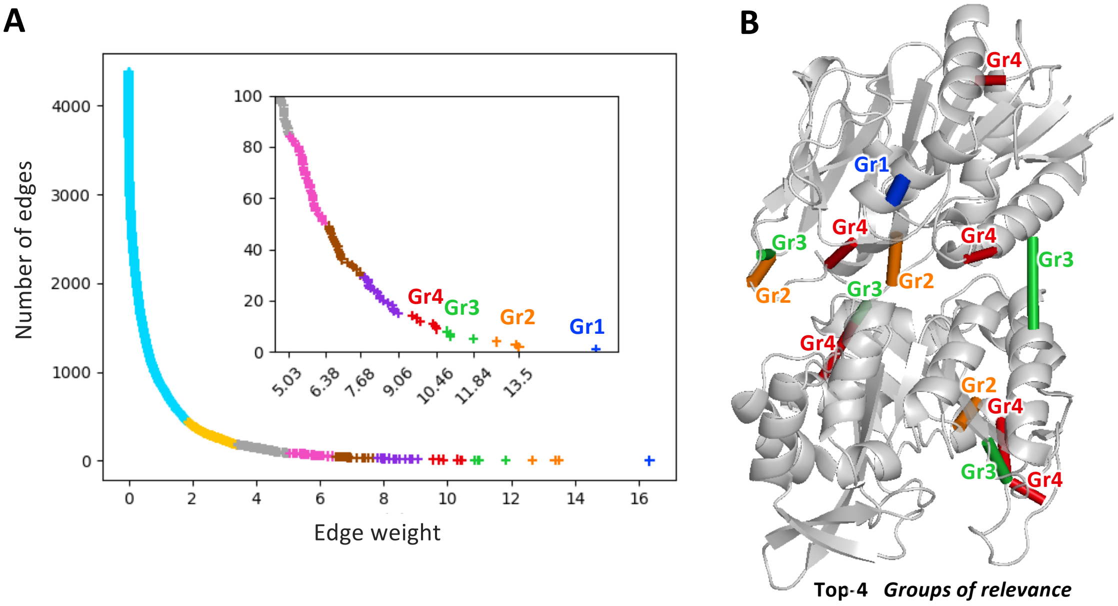

Figure S3: A) Birch clustering of the DPCN graph of IGPS represented within the plot of the number of edges decay as a function of contact weight. Each color of the dots is associated to a different cluster, i.e. to each group of relevance (Gr). The inset zooms in the region of the first seven groups of relevance. B) 3D representation of IGPS including the DCPN graph selection based on the top-four groups of relevance, following the same color scheme of panel A.

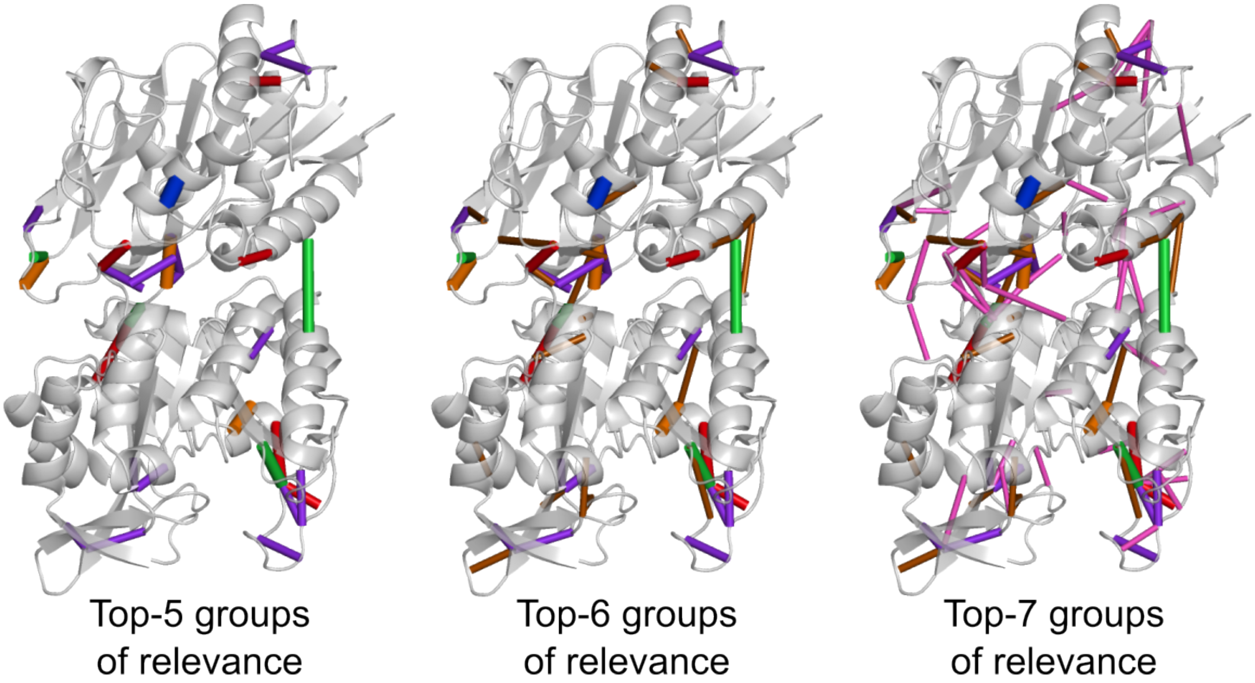

Figure S4: Top-5 to 7 groups of relevance using Birch clustering with no final clustering step ($n_{\text{clusters}} = 11$)

In other words, one can cluster the edges, which are weighted by the number of contacts perturbations, according to their weights, i.e. with edges belonging to the same cluster having similar values of weights. Then, one can put these clusters in an order, going from the first one featuring the largest weights to the last one with the smallest edges. Since these clusters are ranked by weights that refer to the contact perturbations, they represent a list of groups of edges ordered by their relevance in the perturbation contact network, thus they are named "groups of relevance". So, the ordered groups of relevance can be simply interpreted as the clusters of edges present in DPCN ordered by their relevance. Thus, their selection and interpretation does not require any previous knowledge of the proteic systems under investigation.

Birch clustering offers several key advantages for our analysis. Firstly, it is a hierarchical clustering method, allowing for versatile exploration of the data. By producing a set of clusters without setting a fixed number of them, it offers flexibility in the subsequent analysis and interpretation of the results, while guaranteeing that the groups formed are of similar

relevance. Moreover, Birch clustering has been specifically designed to handle large-scale data efficiently and demonstrates robustness against noise. This scalability and noise tolerance further support its applicability to larger systems that are prone to thermal fluctuations.

In Birch clustering, the first output is a set of clusters produced without setting a fixed number of them, with re-clustering that can be subsequently performed using a predefined number of clusters. Here, we have conducted the analysis using the first output from Birch clustering (while re-clustering using 2–3 clusters is also reported in Figure S5 for completeness). Figure S3 shows the counting of edges decay as function of contact weight (previously shown in Fig. 1C) combined with the Birch clustering results. By setting a given number of edges as cutoff, corresponding to the selection of a given threshold weight discussed above (i.e. 50 edges and 6.38 threshold), one of the groups of relevance obtained by clustering is likely cut at an arbitrary point. In contrast, one could exploit the clustering output and analyze the clusters in decreasing order of relevance, stopping at the desired number of clusters.

Overall, the most interesting outcome of the clustering procedure is the appearance of a connection between the residues directly bound to the effector and *loop1* (involving residues fA224, fF227, fH228, fK19, fD11 and fR27), which was not clearly observed in previously analyses of IGPS allostery. However, this connection is dislocated in three different groups of relevance. Indeed, as expected, this analysis does not provide insights on the local propagation of contact perturbations and, thus, straightforward information on the allosteric signaling mechanism. In fact, increasing the number of groups of relevance (e.g. more than the four groups considered so far) will not necessarily provide more information on local propagation of perturbations, while it complicates the analysis due to the quick increase of the total number of edges to be considered (see Figure S3).

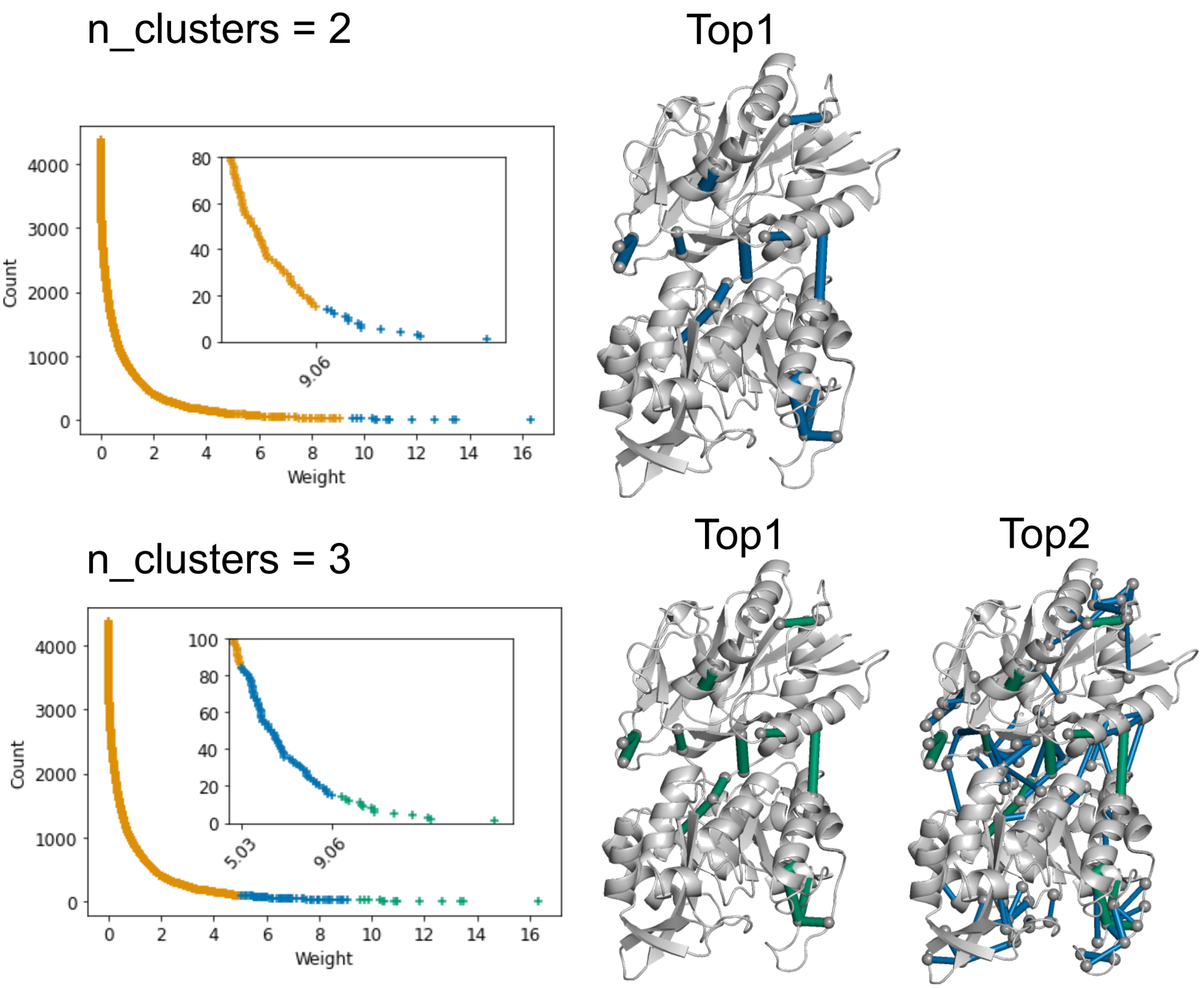

Figure S5: (*top*) Birch clustering with 2 clusters displaying the top cluster on the protein. (*bottom*). Birch clustering with 3 clusters displaying the top-one and top-two cluster on the protein.

Detailed CCA analysis for bacterial IGPS

Two of the largest components in the final CC structure relate to the alteration of motion in *loop1*, representing the first contact perturbations upon effector binding (i.e., components 1 and 9). They show clearly how the perturbations at the effector binding site preferentially propagates along the sideR of HisF, as previously reported.[3–5] Then, the contact perturbations reach the HisF/HisH interface, with two CCs (namely 4 and 8) being associated with the rearrangements of contacts between surface-exposed residues, involving known alterations of salt-bridges connecting the $f\alpha 2$, $f\alpha 3$, $h\alpha 1$ and $h\alpha 4$ helices.[3,4] These interface

contacts, perturbed by effector binding, have two-fold major effect in HisH. On one side, they alter the breathing motion, thus affecting contacts at the sideL of HisH (and also at the interface), belonging to CCs 2,3 and 6. On the other side, they induce significant changes in the HisH active site, involving the known hydrogen-bonding network between the Ω-*loop* and the 49-PGVG sequence and the consequent flipping of the oxyanion strand. Notably, only one among the nine CCs with $d>2$, i.e. the component 7, could not be attributed to secondary structure elements that are strictly associated to the IGPS allosteric mechanism. However, this outlier component involves the highly mobile C-terminus of HisH (see Figure 2 in ref 4) that is prone to large thermal fluctuations and thus to potential overestimation of the contact perturbations.

Table S1: Secondary structure elements of the IGPS complex involved in the CCs featuring a diameter (d) larger than 2. The labels of CCs (1-9) follow the decreasing order of d. The size and vanishing point (v.p.) of each component are also reported.

**CC**	**d**	**v.p.**	**Size**	**Secondary structure elements**
**1**	7	13.38	23	loop1, fα1, fβ1, fβ5-fα5, fβ8-fα8
**2**	6	10.46	15	fα3-fβ4, fα4-fβ5, fα6-fβ7, fβ4, fβ6, hβ6-hβ7, hβ7, hβ8', hβ9
**3**	4	13.51	6	hβ10, hβ6-hβ7, hβ8, hβ9-hβ10
**4**	4	10.97	9	fα2, fα2-fβ3, hα1, hα4
**5**	3	16.34	6	oxyanion strand, hα2, Ω-loop
**6**	3	12.68	9	fC-term, fN-term, fα7, fα8, hβ8'
**7**	3	9.72	6	hC-term, hα4, hβ10-hβ11, hβ11, hβ4
**8**	3	9.56	4	fα2, fα3, fα3-fβ4, hα1, Ω-loop
**9**	3	5.39	4	loop1, fβ6-fα6, fβ7-fα7

After showing how the CCA can effectively detect the propagation of the atomic contact perturbations associated to an allosteric signal, in the following we discuss the additional insights that this method provided for the characterization of allostery in the IGPS complex. As shown in Figure S6, the CCA applied to the DPCN graph yields CCs (1, 4, 5, 8 and 9) that highlight relevant perturbations in both the effector binding site (see Figure S6A) and the active site (see Figure S6B). In particular, the CCs in the effector site (1 and 9) clearly highlight perturbations that start from residues bound to the PRFAR effector (fA224 and fA204 interacting with the glycerol phosphate group, fS144 interacting with the imidazole-

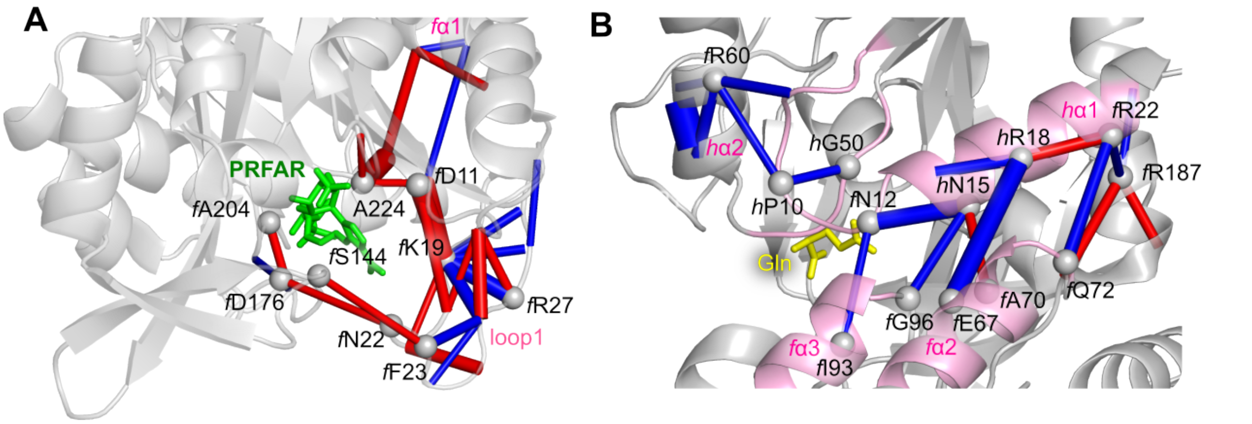

Figure S6: Detailed view of the DPCN of IGPS focusing on the CCs nearby the effector (A) and active (B) sites, highlighting the main amino acid residues (black labels) and secondary structures (in pink) involved in the allosteric signal propagation.

carboxamide group, and fD176 with the hydroxyl group at the glycerol side) and reach the *loop1* (in particular residues fN22 and fF23), through the fS144-fF23 and fD176-fN22 contacts. These contacts increase upon effector binding, in line with the closing of *loop1* once PRFAR enters in its binding site. Notbaly, the CCA indicates that almost all residues in *loop1* are interconnected within the CC with the largest size and diameter (i.e. component 1), and these perturbations add up to those in the $f\beta 8$-$f\alpha 8$ turn (fA224), reaching the $f\alpha 1$ helix, see Figure S6A. At the HisF/HisH interface and in the HisH active site, the CCA captures quite effectively the most relevant allosteric effects, including the critical fE67-hR18 salt-bridge that involve both $f\alpha 2$ and $f\alpha 1$ helices and the hN15-hN12 contact for the $f\alpha 3$-Ω-*loop* interaction. Interestingly, these known alterations in the active sites propagate, as expected, from the Ω-*loop* to the 49-PGVG sequence, see the hP10-hG50 contact, but are also directly connected to the hE56-hR59 salt-bridge in the $h\alpha 2$, which features the largest weight (i.e. contact perturbation) in the whole DPCN graph. The weakening of the hP10-hG50 interaction is obviously associated to the recognized allosteric event of the hP10-hV51 hydrogen-bond breaking (causing the oxyanion strand flip) and the CCA indicates that it is directly related to the breaking of the hE56-hR59 interaction. This suggests that point mutations of the hE56 and/or hR59 residues might significantly alter the activity of the

IGPS complex.

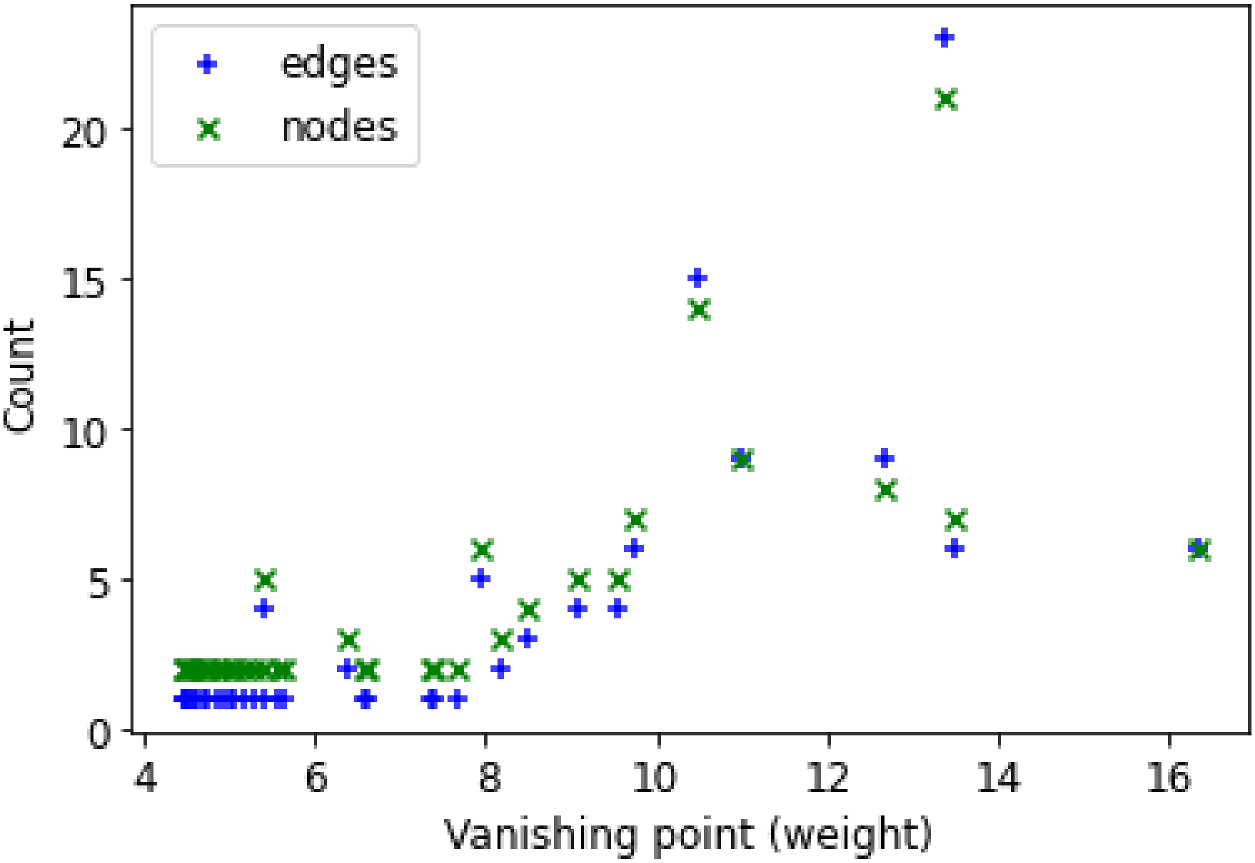

Figure S7: Scatter plot of the size (number of edges) and order (number of nodes) of each component against their vanishing point. There is a tendency of big vanishing points to create big components albeit not a complete correlation.

References

(1) Pedregosa, F. et al. Scikit-learn: Machine Learning in Python. *J. Mach. Learn. Res.* **2011**, *12*, 2825–2830.

(2) Zhang, T.; Ramakrishnan, R.; Livny, M. BIRCH: an efficient data clustering method for very large databases. *ACM SIGMOD Rec.* **1996**, *25*, 103–114.

(3) Gheeraert, A.; Pacini, L.; Batista, V. S.; Vuillon, L.; Lesieur, C.; Rivalta, I. Exploring

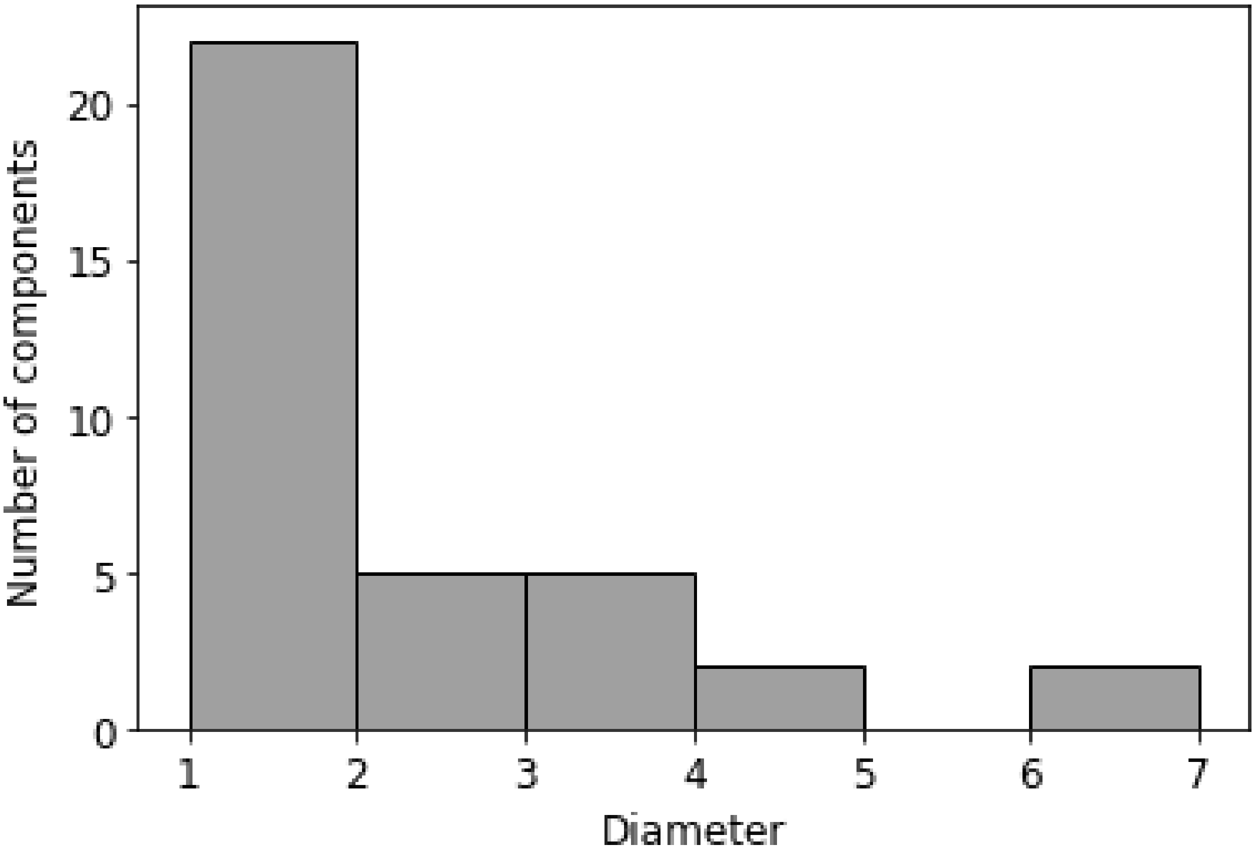

Figure S8: Distribution of the diameters in the final components. 22 components have a diameter of 1 (thus consisting of a single edge) while 5 have a diameter of 2 (trivial examples of propagations). The ninth major component have a diameter bigger or equal than 3.

allosteric pathways of a v-type enzyme with dynamical perturbation networks. *J. Phys. Chem. B* **2019**, *123*, 3452–3461.

(4) Rivalta, I.; Sultan, M. M.; Lee, N.-S.; Manley, G. A.; Loria, J. P.; Batista, V. S. Allosteric pathways in imidazole glycerol phosphate synthase. *Proc. Natl. Acad. Sci.* **2012**, *109*, E1428–E1436.

(5) Negre, C. F.; Morzan, U. N.; Hendrickson, H. P.; Pal, R.; Lisi, G. P.; Loria, J. P.; Rivalta, I.; Ho, J.; Batista, V. S. Eigenvector centrality for characterization of protein allosteric pathways. *Proc. Natl. Acad. Sci.* **2018**, *115*, E12201–E12208.
